# Anatomical and Physiological Science among the Ancient Egyptians

**Published:** 1901-01

**Authors:** 


### ANATOMICAL AND PHYSIOLOGICAL SCIENCE AMONG THE ANCIENT EGYPTIANS.

THIS subject was presented before the Anthropological Society of Paris, by M. Garnaud, who said that it had long been the general belief that the ancient Egyptians had never acquired any positive knowledge of the world and of life. But this opinion is no longer tenable, as the Greeks owed much of their first knowledge to them, and we now possess manuscripts to which are consigned the secrets and receipts of their craftsmen which give evidence of a profound knowledge of the chemical properties of a number of bodies and their combinations. Very ancient legends affirm that the God Thoth had studied human anatomy and that the Son of Menes had taught it to men. Prof. Flinders Petrie has recently discovered a papyrus, which, according to him, is a copy of ancient documents found at the feet of the statue of the God Thoth. It dates to 1550 B. C., and contains what the Egyptians knew of the human body. Garnaud concludes that these anatomical and physiological notions reach back into even more remote antiquity. In the pre-Pharaonic era embalmment was not practiced, and in certain tombs the body was placed after the soft parts had been detached from the skeleton. He further concludes that the Egyptians practiced upon their dead a sort of ritual dissection, and that from this epoch the anatomical ideas of the Egyptians are to be dated. These remained in a state of immobility without realizing any progress, and their civilization suffered a long eclipse comparable to that of our dark ages. Life, according to their primitive notion, was determined by the *pneuma*, a sort of breath, an aerial spirit which entered the body by the mouth, nose and ears and became mixed with the blood of the heart. All rejected vapors, worked over and corrupted *pneuma* came from the heart. The heart communicates with the stomach; it is felt in all parts of the body. The Egyptians well understood the pulse. They represented the heart as a pot with two handles. Sound, which entered at the ears, was also air (they knew nothing of the tympanum), a pneumatic emanation from the object which penetrated the body. The name, the noun, the verb, had then an objective existence. Nothing was known of the brain; it played no part except in the pharmacopœia. Remedies were regarded as applications of the pneuma, the spirit of things. The endeavor was always to force into the body good pneuma, like that of incense. The womb was a wild animal, and many kinds of pneuma were conveyed through the vagina. The Egyptians pretended to be able to foretell the sex of unborn children. They placed in water to sprout grains of wheat and grains of barley. If the wheat sprouted first, a boy was announced; if the barley prevailed, a girl would be born. If neither germinated, the woman was certain to abort.

---

DR. BERNARD WOLFF has acquired Dr. Dunbar Roy's interest in THE JOURNAL-RECORD. Dr. Roy's effective cooperation will be sorely missed.

